# Comparative Evaluation of Feature Extractors, Aggregation Strategies, and Classification Hierarchies for Ovarian Cancer Subtype Classification in Whole Slide Images

**DOI:** 10.3390/diagnostics16101570

**Published:** 2026-05-21

**Authors:** Ho Jung Song, You Sang Cho, Yong Suk Kim

**Affiliations:** 1 Department of Medical Engineering, Konyang University, 158 Gwanjeo-dong-ro, Seo-gu, Daejeon 32992, Republic of Korea; songhj6692@gmail.com (H.J.S.); davidecho@naver.com (Y.S.C.); 2 Department of Artificial Intelligence, Konyang University, 158 Gwanjeo-dong-ro, Seo-gu, Daejeon 32992, Republic of Korea

**Keywords:** ovarian cancer classification, multiple instance learning, whole slide image analysis, feature extractor comparison, hierarchical classification, computational pathology

## Abstract

**Background/Objectives**: Multiple instance learning (MIL) is widely used for automated classification of epithelial ovarian cancer subtypes from whole slide images (WSIs), but the relative contributions of feature extractor, aggregation strategy, and classification framework (flat vs. hierarchical) choices remain unclear under severe class imbalance. **Methods**: We evaluated 36 configurations on 510 WSIs from the UBC-OCEAN dataset using stratified five-fold cross-validation, comparing three pathology foundation models (Phikon-v2, CTransPath, UNI), six aggregators (mean/max pooling, ABMIL, CLAM-SB, DSMIL, DTP-TransMIL), and two classification strategies. Pathologist-annotated WSIs assessed attention map interpretability. **Results**: Feature extractor selection contributed substantially more variance than aggregator choice. Cascade balanced accuracy ranged from 0.538 (Phikon-v2) to 0.925 (UNI); CTransPath (~32 K pretraining WSIs) reached 0.870, exceeding Phikon-v2 (~58 K WSIs) and approaching UNI (~100 K+ WSIs), indicating that pretraining objective and architecture contribute as substantially as scale. The hierarchical cascade consistently improved high-grade serous carcinoma (HGSC) recall across all six evaluated configurations (+0.073 to +0.530), detecting 206 of 217 cases (0.949) with UNI max pooling. Quantitative spatial alignment analysis confirmed that both stronger feature extractors—CTransPath and UNI—generated significantly more spatially structured attention distributions than Phikon-v2 (paired Wilcoxon, *p* = 0.008 and *p* = 0.032, respectively). **Conclusions**: Feature extractor choice contributed more variance than aggregator selection, with the largest gap between Phikon-v2 and stronger extractors. Hierarchical cascades consistently improved HGSC recall across all configurations.

## 1. Introduction

Epithelial ovarian cancer (EOC) is the most lethal gynecological malignancy, accounting for approximately 324,000 new cases and 207,000 deaths annually worldwide [1]. EOC is classified into five major histological subtypes: high-grade serous carcinoma (HGSC), endometrioid carcinoma (EC), clear cell carcinoma (CC), low-grade serous carcinoma (LGSC), and mucinous carcinoma (MC). These subtypes differ substantially in their molecular profiles, clinical behaviors, and therapeutic responses [2,3]. HGSC, the most prevalent subtype, is characterized by aggressive progression, frequent TP53 mutations, and sensitivity to platinum-based chemotherapy [4]. In contrast, CC and MC subtypes often demonstrate platinum resistance and require alternative treatment strategies [5]. Accurate histological subtype classification is therefore essential for guiding personalized treatment decisions and improving patient outcomes.

Histopathological examination of hematoxylin and eosin (H&E)-stained tissue sections remains the gold standard for ovarian cancer subtype diagnosis. However, this process is labor-intensive, time-consuming, and subject to considerable inter-observer variability, particularly in distinguishing morphologically similar subtypes such as HGSC and EC [6,7]. The increasing adoption of digital pathology and whole slide image (WSI) scanning has created new opportunities for computational approaches to assist pathologists in this diagnostic task [8]. The analysis of WSIs presents unique computational challenges due to their gigapixel resolution, typically ranging from 50,000 × 50,000 to 200,000 × 200,000 pixels at 20× magnification. Multiple instance learning (MIL) has emerged as the dominant paradigm for WSI classification, treating each slide as a “bag” of patch-level “instances” and learning to aggregate instance-level features into slide-level predictions [9,10].

A standard MIL pipeline for WSI classification involves three sequential design choices. First, a pretrained feature extractor generates fixed-dimensional embeddings for each patch. Recent pathology-specific foundation models such as CTransPath [11], UNI [12], Virchow [13], and Phikon-v2 [14] have substantially improved feature representations over ImageNet-pretrained encoders. Second, an aggregation strategy combines instance-level features into a single slide-level representation. Methods range from simple pooling (mean, max) to attention-based approaches including ABMIL [15], CLAM [16], DSMIL [17], and TransMIL [18]. Third, the classification framework determines whether subtypes are predicted in a single flat multi-class model or through a hierarchical decomposition into sequential binary decisions.

Despite the proliferation of MIL methods, a fundamental question remains insufficiently addressed: what is the relative importance of each pipeline component? While individual studies have proposed increasingly sophisticated aggregation architectures, few have systematically quantified how much each design choice contributes to overall performance. This gap is especially critical in ovarian cancer classification, where severe class imbalance further complicates pipeline optimization. When conventional flat multi-class classification is applied to such imbalanced datasets, models face a critical dilemma: optimizing solely for overall metrics often leads to the paradoxical under-detection (i.e., majority suppression) of the most common and lethal subtype, HGSC, particularly when heavy penalties are applied to minority class errors. Hierarchical classification offers a principled alternative that decomposes the multi-class problem into sequential, clinically motivated binary decisions [19]. In clinical practice, distinguishing HGSC from non-HGSC subtypes is often the primary diagnostic question, as it directly determines the initial treatment pathway [4].

In this study, we present a comparative evaluation of MIL pipeline components for ovarian cancer subtype classification in WSIs. The main contributions of this work are as follows:

(1) Among the aggregators evaluated, we introduce DTP-TransMIL (Dynamic Top-Proportion TransMIL), an extension of TransMIL [18] with dynamic top-proportion patch selection adapted to the wide variation in WSI patch counts (228–34,412 in our cohort). While DTP-TransMIL achieved the strongest performance with weaker features, our broader analysis revealed that simpler aggregators (max pooling) matched or exceeded its performance with stronger features—a finding that itself motivates the comparative framework of this study.

(2) We conduct a comprehensive comparison of 36 experimental conditions (3 feature extractors × 6 aggregators × 2 classification strategies) under strictly identical protocols. We quantify the relative contribution of each component, finding that the choice of feature extractor accounted for substantially more variance (+0.387 in cascade balanced accuracy between Phikon-v2 and UNI) than aggregator selection (±0.053 within each feature space). Notably, CTransPath—pretrained on a smaller corpus than Phikon-v2—achieved performance comparable to UNI, indicating that pretraining objective and architecture contribute as substantially as scale to feature quality.

(3) We demonstrate that a hierarchical two-stage cascade effectively mitigates the under-detection of dominant classes in severely imbalanced WSI cohorts. Across all six configurations evaluated (3 features × 2 aggregators), the cascade improved high-grade serous carcinoma (HGSC) recall by +0.073 to +0.530 over flat five-class classification, with per-class recalls exceeding 0.86 for all five subtypes in the optimal pipeline.

(4) Using pathologist-annotated WSIs, we provide both qualitative and polarity-agnostic quantitative evidence that the spatial interpretability of MIL attention maps is associated with feature extractor quality, with stronger foundation features producing attention patterns that depart significantly from random distributions (paired Wilcoxon *p* < 0.05) compared to weaker features.

## 2. Materials and Methods

**Figure 1:**
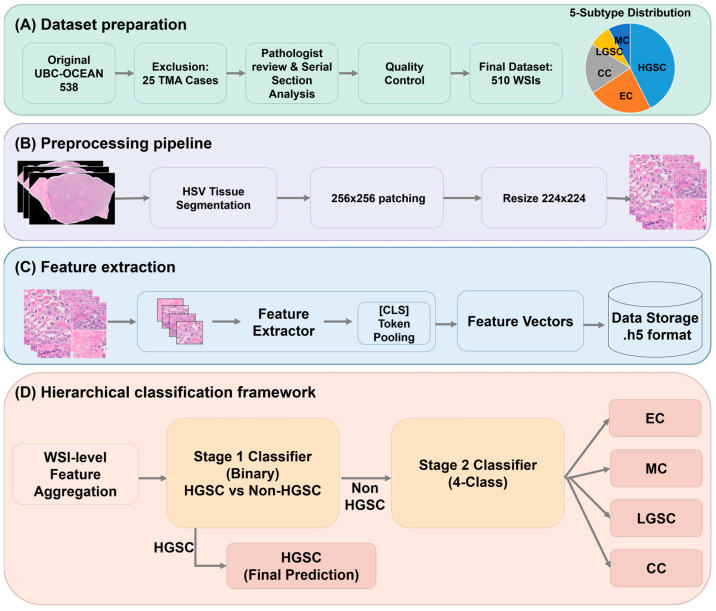
Overview of the proposed computational pathology pipeline for ovarian cancer subtype classification. (**A**) Dataset preparation and quality control process, resulting in a final cohort of 510 whole slide images (WSIs) from the multi-institutional UBC-OCEAN dataset. The pie chart illustrates the imbalanced distribution of the five histological subtypes. (**B**) WSI preprocessing pipeline, including HSV-based tissue segmentation, patch extraction at 256 × 256 pixels, and resizing to 224 × 224 pixels. (**C**) Patch-level feature extraction using three frozen pathology foundation models (Phikon-v2, CTransPath, and UNI) to generate 768-dimensional (CTransPath) or 1024-dimensional (Phikon-v2, UNI) feature vectors. (**D**) The hierarchical two-stage classification framework: Stage 1 separates HGSC from non-HGSC subtypes via binary classification, while Stage 2 classifies the four remaining subtypes among slides routed as non-HGSC.

The overall computational pathology pipeline, including dataset preparation, feature extraction, and the hierarchical classification framework, is illustrated in Figure 1.

### 2.1. Dataset and Preprocessing

This study utilized the UBC Ovarian Cancer subtype classification and Outcome prediction (UBC-OCEAN) dataset, a publicly available collection of histopathological images compiled from over 20 medical institutions [20]. The original training set comprised 538 images, including both WSIs at 20× magnification and tissue microarray (TMA) images at 40× magnification. We excluded all 25 TMA images to retain only WSI-format slides. For slides containing multiple serial tissue sections, a board-certified pathologist reviewed and selected a single representative section. Following automated tissue segmentation and quality control (removal of slides with fewer than 10 tissue patches or severe imaging artifacts), a final cohort of 510 WSIs was established.

The dataset encompasses five EOC subtypes: HGSC (*n* = 217, 42.5%), EC (*n* = 118, 23.1%), CC (*n* = 93, 18.2%), LGSC (*n* = 41, 8.0%), and MC (*n* = 41, 8.0%). The number of tissue patches extracted per slide ranged from 228 to 34,412 (median: 7864), representing a 151-fold variation in tissue area. The dataset characteristics are summarized in Table 1.

Tissue regions were identified through automated segmentation: slides were downsampled by a factor of 32, converted to the HSV color space, and binarized by thresholding the saturation channel at 15. Morphological closing followed by opening was applied using an elliptical structuring element of size 7 × 7 with two iterations each. Patches were extracted using a non-overlapping grid of 256 × 256 pixels at the original resolution. Only patches with a tissue proportion exceeding 20% were retained.

### 2.2. Feature Extraction

Patch-level feature representations were extracted using three pathology foundation models in a frozen configuration (no fine-tuning):

Phikon-v2 [14]: A histopathology-specific foundation model based on the Vision Transformer-Large (ViT-L) architecture, pretrained via self-supervised learning on approximately 58,000 WSIs. The [CLS] token output was extracted as a 1024-dimensional feature vector.

CTransPath [11]: A histopathology-specific foundation model based on the Swin Transformer Tiny (Swin-T) architecture, pretrained on approximately 32,000 WSIs from TCGA and PAIP via semantically relevant contrastive learning (SRCL). The global average-pooled feature was extracted as a 768-dimensional feature vector.

UNI [12]: A general-purpose pathology foundation model based on the ViT-L architecture, pretrained on over 100,000 WSIs from diverse tissue types. The [CLS] token output was extracted as a 1024-dimensional feature vector.

The three feature extractors produced embeddings of differing dimensionality (768 for CTransPath; 1024 for Phikon-v2 and UNI). The corresponding input dimensions were used for each aggregator, with all other architectural choices held constant to ensure a fair comparison across configurations. All patches were resized to 224 × 224 pixels and standardized using ImageNet mean and standard deviation values.

### 2.3. MIL Aggregation Methods

Six aggregation methods were implemented and evaluated under identical experimental conditions. Mean and Max Pooling, ABMIL, CLAM-SB, and DSMIL utilized all extracted patches without sampling, while DTP-TransMIL applied dynamic top-25% selection as described below. This design isolated patch sampling as a controlled variable specific to DTP-TransMIL.

Mean and Max Pooling: Non-parametric baselines that aggregate all patch representations by computing the element-wise average or maximum, respectively.

ABMIL [15]: Computes a weighted sum of patch representations using a gated attention mechanism with tanh and sigmoid activations.

CLAM-SB [16]: Extends attention-based aggregation by introducing instance-level clustering constraints on the highest- and lowest-attention patches.

DSMIL [17]: Adopts a dual-stream architecture combining an instance-level classifier with a bag-level aggregator, fusing both streams for the final prediction.

DTP-TransMIL (Dynamic Top-Proportion TransMIL), introduced in this work, is a Transformer-based aggregator that extends TransMIL [18] with dynamic top-proportion patch selection, adaptively scaling the number of selected patches based on each slide’s total tissue area. The model selects the top 25% of patches (bounded by $K_{min}=100$ and $K_{max}=2000$), re-weights them by sigmoid-activated attention scores, and processes the sequence through a two-layer Transformer encoder with 8 attention heads. The [CLS] token output serves as the slide-level representation.

The three DTP-TransMIL hyperparameters (selection ratio = 0.25, *K_min_* = 100, *K_max_* = 2000) were chosen to balance representational coverage and computational tractability across the wide patch-count range of the cohort (228–34,412 patches per slide, median 7864; Table 1). The proportional ratio of 0.25 ensures that the number of selected tokens scales with slide size, while *K_min_* = 100 prevents underrepresentation on small slides—without this floor, slides with N < 400 patches (which constitute ~5.5% of the cohort) would receive fewer than 100 tokens, insufficient for stable Transformer attention. *K_max_* = 2000 was set based on two considerations. First, the self-attention complexity of the Transformer encoder scales as O(K^2^) in memory, and K = 2000 is the largest value that fits within 16 GB GPU memory alongside the per-token 512-dimensional embeddings during training with batch size 1. Second, the saturation point N* = *K_max_*/0.25 = 8000 patches places approximately half of the cohort (slides with N < 8000) into the proportional regime, where token selection scales naturally with slide size, and the other half into the ceiling regime (*K_eff_* = 2000), where the most informative 2000 tokens are retained regardless of slide size.

All models shared the same input projection structure (768→512 dimensions for CTransPath, 1024→512 dimensions for Phikon-v2 and UNI, with ReLU activation and dropout) and classifier architecture. Critical hyperparameters—including learning rate ($1\times 10^{-4}$), weight decay ($1\times 10^{-4}$), total training epochs (150), and early stopping patience (30 epochs)—were held constant across all methods.

The six aggregators differ markedly in computational complexity, as summarized in Table 2. MeanPool and MaxPool are the lightest (0.66 M parameters and 8.3 GFLOPs at N = 7864), with the input projection fc1 accounting for ~80% of their parameters. ABMIL, CLAM-SB, and DSMIL are 20% heavier (~0.79 M parameters), with the additional cost concentrated in the gated attention modules (V and U), which contribute ~33% of total parameters. DTP-TransMIL is substantially more expensive (4.997 M parameters, 36.5 GFLOPs, 5.7× the inference time of MeanPool); 84% of its parameters reside in the two-layer Transformer encoder, while the input projection accounts for only 10.5%. Despite this difference, all aggregators complete a single-slide forward pass in under 3 ms on a single GPU at the median patch count, indicating that aggregator complexity is not a practical bottleneck in clinical-throughput scenarios.

### 2.4. Classification Strategies and Experimental Setup

We compared two overarching classification frameworks:

Flat Five-Class Classification: A single model directly predicts all five subtypes from each WSI.

Hierarchical Two-Stage Cascade: Stage 1 employs a binary classifier to distinguish HGSC from non-HGSC subtypes. Stage 2 classifies the four remaining subtypes (EC, CC, LGSC, MC) among slides routed as non-HGSC. The two stages were trained independently with separate model instances. At inference, the Stage 1 threshold was determined using Youden’s J statistic (maximizing sensitivity + specificity − 1) on each validation fold. Slides classified as HGSC received their final label immediately, while remaining slides were processed by the Stage 2 model.

Model Training and Evaluation: Model performance was evaluated using stratified five-fold cross-validation with a fixed random seed. For the hierarchical approach, Stage 1 stratification was based on binary labels across 510 WSIs, while Stage 2 stratification followed the four-class distribution among 293 non-HGSC specimens. Fold assignments were strictly held constant across all methods. Focal loss [21] with γ=2.0 and inverse-frequency class weights (exponent 1.5) was employed to address class imbalance. All models were optimized using Adam with cosine annealing and linear warmup (15 epochs), mixed-precision (FP16) arithmetic, and gradient clipping (max norm 1.0). Early stopping monitored AUROC for Stage 1 and balanced accuracy for Stage 2. For end-to-end cascade evaluation, per-class precision, recall, and F1-score were additionally reported using out-of-fold predictions from both stages.

The focal loss γ = 2.0 and class weight exponent *p* = 1.5 were selected a priori based on prior literature [21]. To assess sensitivity to these choices, we conducted a 3 × 3 grid search (γ ∈ {1.0, 2.0, 3.0} × *p* ∈ {1.0, 1.5, 2.0}) on the Stage 1 task using UNI features and max pooling, repeated across all five cross-validation folds (45 runs total). Performance was highly stable across the nine configurations: AUROC ranged 0.981–0.984 (span 0.0024), HGSC recall ranged 0.940–0.959 (span 0.019), and balanced accuracy ranged 0.945–0.957 (span 0.012). Inter-fold variability within each configuration exceeded inter-configuration variability by a factor of 17 for AUROC and 7 for balanced accuracy, indicating that performance is essentially insensitive to these hyperparameters within the tested ranges.

Statistical Analysis: The Friedman test assessed overall differences among aggregators, followed by pairwise one-sided Wilcoxon signed-rank tests with Holm–Bonferroni correction. Given the limited statistical power of five-fold evaluation (minimum achievable *p* = 0.031), Cohen’s d was reported as a complementary measure of effect size.

### 2.5. Spatial Interpretability Analysis

To assess whether model attention aligns with histologically meaningful regions, we conducted a qualitative analysis comparing attention heatmaps against pathologist annotations. A board-certified pathologist with over 20 years of experience annotated a subset of 256 WSIs comprising HGSC, CC, and EC cases. The annotation targets were deliberately mixed: in certain slides, the annotations delineate malignant epithelial regions, whereas in others, they highlight normal tissue and stromal areas. Attention scores generated by the DTP-TransMIL aggregator were normalized and spatially mapped back to WSI coordinates for all three feature extractors (Phikon-v2, CTransPath, and UNI). These heatmaps were qualitatively compared to determine how feature extractor quality influences the spatial interpretability and pathological reliability of MIL attention mechanisms.

To complement the qualitative analysis, we computed two polarity-agnostic quantitative metrics on each annotated WSI. The Normalized Attention Alignment Ratio (NAAR) was defined as the ratio of mean attention scores within annotated regions to mean attention scores over the entire valid tissue area. Values close to 1.0 indicate spatial distributions indistinguishable from random; |NAAR − 1.0| measures the deviation from random regardless of whether the annotated region depicts malignant or non-malignant tissue, accommodating the mixed annotation design described above. The secondary metric, Attention Mass Ratio (AMR), was computed at the top 10%, 25%, and 50% attention thresholds, with deviation from the random expectation (baseline = annotated area fraction) reported as |AMR − baseline|. For each feature extractor, attention scores were extracted using the corresponding Stage 1 (HGSC vs. non-HGSC) DTP-TransMIL model under five-fold out-of-fold evaluation. Statistical comparisons between feature extractors used paired Wilcoxon signed-rank tests on |NAAR − 1.0|, restricted to slides for which all three features were evaluable.

## 3. Results

### 3.1. Impact of Feature Extractor on Classification Performance

To quantify the relative contribution of the feature extractor to overall pipeline performance, we evaluated all six aggregation methods under three feature extraction conditions (Phikon-v2, CTransPath, and UNI) using the hierarchical two-stage classification framework.

As shown in Table 3, the choice of feature extractor produced substantial and consistent performance gradients across all aggregators and evaluation metrics. In Stage 1 (HGSC vs. non-HGSC), the mean AUROC across the six aggregators was 0.679 with Phikon-v2, 0.953 with CTransPath, and 0.978 with UNI. In Stage 2 (EC/CC/LGSC/MC), the corresponding mean balanced accuracy was 0.584, 0.903, and 0.931, respectively. Notably, CTransPath—pretrained on a smaller corpus (~32,000 WSIs) than Phikon-v2 (~58,000 WSIs)—substantially exceeded Phikon-v2 across all metrics and approached UNI (~100,000+ WSIs), indicating that pretraining objective and architecture contribute as substantially as scale to feature quality. Even the weakest aggregator with UNI features (mean pooling, Stage 2 balanced accuracy = 0.904) substantially outperformed the strongest aggregator with Phikon-v2 features (DTP-TransMIL, Stage 2 balanced accuracy = 0.634).

In contrast, the performance variance attributable to aggregator selection within each feature space was markedly smaller. With Phikon-v2, the best-to-worst aggregator gap in Stage 2 balanced accuracy was 0.130 (DTP-TransMIL: 0.634 vs. mean pooling: 0.504). With CTransPath, this gap narrowed to 0.041 (max pooling: 0.926 vs. mean pooling: 0.885). With UNI, the gap was 0.053 (max pooling: 0.957 vs. mean pooling: 0.904), substantially smaller than under Phikon-v2. This convergence indicates that stronger feature representations substantially reduce the relative importance of the aggregation mechanism.

**Figure 2:**
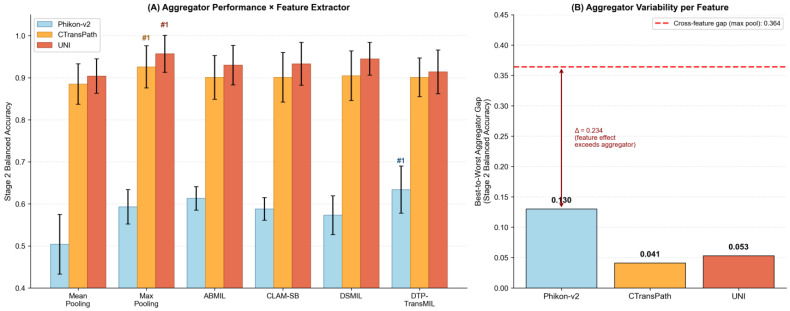
Aggregator performance comparison across three feature extractors (Stage 2, five-fold cross-validation). (**A**) Grouped bar plot of Stage 2 balanced accuracy for six MIL aggregators under three feature extractors (Phikon-v2, CTransPath, UNI). The best aggregator per feature extractor is marked with “#1”: with Phikon-v2, DTP-TransMIL ranks first; with both CTransPath and UNI, max pooling ranks first—demonstrating a feature-quality-driven shift in optimal aggregator. Error bars represent standard deviation across five folds. (**B**) Best-to-worst aggregator gap (range of Stage 2 balanced accuracy across six aggregators) per feature extractor. The gap was substantially smaller under stronger features (CTransPath: 0.041; UNI: 0.053) compared with Phikon-v2 (0.130), indicating that stronger features substantially reduce the relative impact of aggregator choice. The red dashed line shows the cross-feature gap with max pooling (UNI − Phikon-v2 = 0.364), illustrating that feature extractor variability (Δ = 0.234 above the largest within- feature gap) substantially exceeds within-feature aggregator variability.

Furthermore, the ranking of optimal aggregators shifted between feature conditions (Figure 2). With Phikon-v2, DTP-TransMIL—the most architecturally complex method—consistently achieved the highest performance in both stages (Stage 1 balanced accuracy: 0.696; Stage 2 balanced accuracy: 0.634). With CTransPath, DTP-TransMIL retained the top rank in Stage 1 (balanced accuracy: 0.918), while max pooling achieved the highest Stage 2 balanced accuracy (0.926). With UNI, max pooling—the simplest non-parametric aggregator—achieved the highest Stage 2 balanced accuracy (0.957), while DTP-TransMIL ranked fifth (0.914). This progressive shift suggests that the benefit of sophisticated aggregation is inversely related to feature quality: when individual patch embeddings are weakly discriminative, attention mechanisms provide substantial compensatory gains; however, when features are already strongly discriminative, simpler aggregation suffices and additional complexity may introduce overfitting without meaningful performance benefits.

### 3.2. Aggregator Comparison Under UNI Features

Given that UNI features yielded substantially superior performance, we conducted a detailed statistical analysis of aggregator differences using UNI as the primary feature extractor. In Stage 1, all aggregators achieved high discriminative performance, with AUROC values ranging from 0.971 (mean pooling) to 0.982 (max pooling). The narrow performance range (ΔAUROC = 0.011) indicates that binary HGSC discrimination is a highly tractable task under strong feature representations. In Stage 2, greater performance separation emerged. Max pooling achieved the highest balanced accuracy (0.957 ± 0.044), followed by DSMIL (0.945 ± 0.039), CLAM-SB (0.933 ± 0.051), ABMIL (0.930 ± 0.047), DTP-TransMIL (0.914 ± 0.052), and mean pooling (0.904 ± 0.041). The Friedman test revealed a statistically significant difference among aggregators for Stage 2 balanced accuracy (χ^2^ = 20.55, *p* = 0.001), confirming that aggregator selection remains a meaningful design choice.

As shown in Table 4, no individual pairwise comparison reached statistical significance at the corrected α = 0.05 level after Holm–Bonferroni adjustment. This outcome is attributable to the constrained statistical power inherent to five-fold evaluation: with only five paired observations, the minimum achievable raw *p*-value is 0.031, which exceeds the corrected significance threshold. However, Cohen’s d values were consistently large, ranging from 0.55 (vs. DSMIL) to 4.91 (vs. mean pooling), with four of the five comparisons exceeding the conventional threshold for a large effect (*d* > 0.8). The largest effects were observed against mean pooling (*d* = 4.91, Δ = +0.053) and DTP-TransMIL (*d* = 2.88, Δ = +0.043), indicating that max pooling achieved not only higher mean performance but also more consistent fold-level improvements.

### 3.3. Hierarchical Cascade Versus Flat Classification

To evaluate the clinical utility of the hierarchical framework, the end-to-end cascade performance was compared against a flat five-class baseline.

As shown in Table 5, the hierarchical cascade consistently improved HGSC recall over flat classification across all six evaluated configurations (three feature extractors × two aggregators), with improvements ranging from +0.073 (UNI + max pooling) to +0.530 (Phikon-v2 + DTP-TransMIL). With CTransPath features, the improvements were +0.143 for max pooling (0.917 vs. 0.774) and +0.115 for DTP-TransMIL (0.903 vs. 0.788), confirming that the cascade benefit generalizes across feature extractors of varying pretraining scale. With UNI max pooling, the cascade also achieved higher overall balanced accuracy (0.925 vs. 0.915), demonstrating that the hierarchical approach can improve both HGSC detection and overall classification. In two configurations, however, the flat classifier achieved a marginally higher overall balanced accuracy than the cascade (UNI + DTP-TransMIL: 0.882 vs. 0.865, Δ = −0.017; CTransPath + max pooling: 0.876 vs. 0.870, Δ = −0.006). This reversal is attributable to cascade error propagation: Stage 1 misclassifications of non-HGSC cases as HGSC (false positives) removed them from Stage 2 consideration, reducing non-HGSC recall. Nevertheless, even in these conditions, the cascade maintained substantial HGSC recall advantages (+0.074 and +0.143, respectively), underscoring that the hierarchical benefit is specific to majority-class detection rather than overall balanced accuracy.

Table 6 presents the per-class performance of the optimal pipeline configuration. The cascade correctly identified 206 out of 217 HGSC cases through the dedicated Stage 1 binary classifier, with only 11 HGSC cases misclassified as non-HGSC (false negatives) and 14 non-HGSC cases incorrectly routed as HGSC (false positives). All five subtypes achieved recalls exceeding 0.86, with CC (0.957) and MC (0.951) demonstrating particularly strong detection.

**Figure 3:**
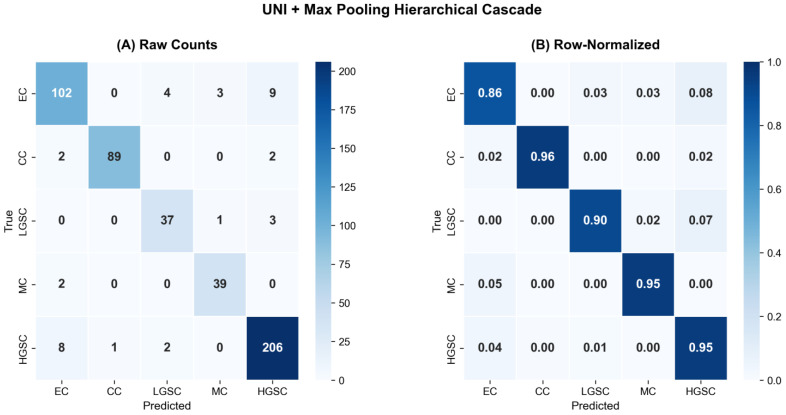
Confusion matrices for the end-to-end hierarchical cascade evaluation using the optimal pipeline configuration (UNI + max pooling). (**A**) Raw counts. (**B**) Row-normalized proportions. The cascade correctly identified 206 of 217 HGSC cases (recall = 0.949). The most frequent misclassification involved EC cases, consistent with the known morphological overlap between EC and HGSC.

The confusion matrices (Figure 3) reveal the error distribution within the cascade. The most common misclassification involved EC cases being predicted as other subtypes, consistent with the known morphological overlap between EC and HGSC [7]. LGSC exhibited the lowest precision (0.860), reflecting occasional confusion with EC and CC—subtypes that can present similar architectural patterns in certain histological contexts.

The flat classifier exhibited a paradoxical pattern: despite achieving competitive overall balanced accuracy (0.915 with UNI max pooling), it detected only 190 of 217 HGSC cases, failing to identify 27 patients with the most clinically aggressive subtype. This phenomenon—majority suppression—arises from the negative interaction between inherent class imbalance and aggressive class-weighting strategies: in a five-class setting, simultaneously upweighting four minority subtypes effectively suppresses the model’s capacity to correctly classify the most prevalent class. The hierarchical approach circumvents this by dedicating Stage 1 entirely to HGSC detection as an explicit binary objective. The consistency of the HGSC recall improvement across all six evaluated configurations (range: +0.073 to +0.530) indicates that this benefit is architecture-agnostic, driven by the structural decomposition itself rather than any specific aggregator-feature combination.

### 3.4. Spatial Interpretability of Attention Maps

To critically assess whether model attention aligns with histologically meaningful regions, we compared attention heatmaps generated by the DTP-TransMIL aggregator using all three feature extractors (Phikon-v2, CTransPath, and UNI) against pathologist annotations across three representative cases: HGSC (slide 2097), CC (slide 7482), and EC (slide 2906_part2).

**Figure 4:**
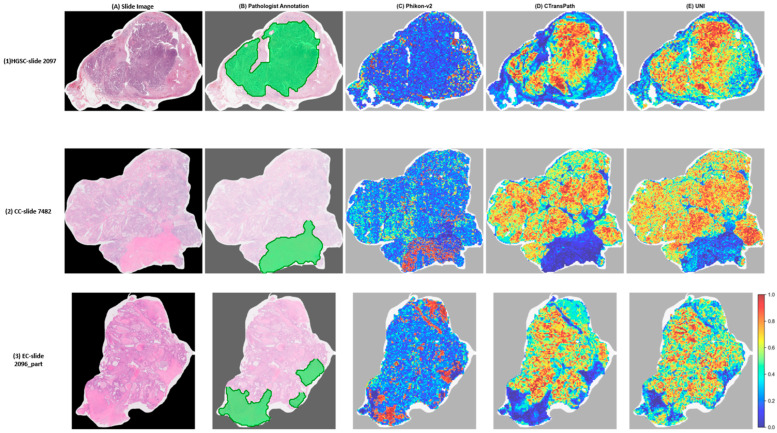
Qualitative comparison of spatial attention patterns across three feature extractors against pathologist annotations. Three representative cases are organized by row—(1) HGSC (slide 2097), (2) CC (slide 7482), and (3) EC (slide 2096_part)—and five panels are shown by column: (**A**) slide image (H&E whole-slide image), (**B**) pathologist annotation (green overlay), (**C**) Phikon-v2-based attention heatmap, (**D**) CTransPath-based attention heatmap, and (**E**) UNI-based attention heatmap. Each cell is therefore uniquely identified by its row–column index (e.g., (1A), (2C), (3E)). In all heatmaps (columns C–E), attention scores are normalized to [0, 1]; warm colors (red) indicate high attention and cool colors (blue) indicate low attention, as shown by the color bar at the bottom right. In the HGSC case (row 1), the annotation delineates malignant epithelium; in the CC and EC cases (rows 2 and 3), the annotations highlight normal tissue and stromal regions. Phikon-v2 attention maps (column C) exhibit spatially diffuse, near-random patterns across all three cases, whereas both CTransPath (column D) and UNI (column E) demonstrate pathologically meaningful spatial structure. Most notably, in the CC case (row 2), both CTransPath and UNI assign their lowest scores to the annotated normal/stromal region, indicating active discrimination between tumor and non-malignant tissue.

Two fundamentally distinct spatial patterns emerged (Figure 4). Phikon-v2-based attention maps (column C) exhibited spatially diffuse, near-random distributions across all three subtypes, with no discernible correspondence to underlying tissue morphology. This pattern is consistent with the low feature discriminability observed in the quantitative evaluation: when individual patch embeddings carry limited subtype-specific information, the attention mechanism lacks a meaningful signal gradient to guide spatial allocation, resulting in uninformative distributions.

In contrast, CTransPath-based (column D) and UNI-based (column E) attention maps demonstrated pronounced spatial structure with clearly delineated regions. The most striking observation appeared in the CC case (Figure 4, middle row): the pathologist annotated a normal/stromal region (green overlay in column B), and both CTransPath and UNI attention maps assigned their lowest scores (dark blue) precisely to this annotated region, while concentrating high attention (warm colors) on the surrounding tumor tissue. This spatial anti-correlation between pathologist-annotated normal regions and model attention indicates that the classifier actively discriminated between tumor and non-malignant tissue.

Similarly, in the HGSC case (Figure 4, top row), CTransPath and UNI attention concentrated on specific tissue regions with clear spatial boundaries, in contrast to the diffuse Phikon-v2 pattern. In the EC case (Figure 4, bottom row), both stronger features preferentially targeted regions distinct from the annotated areas, exhibiting a spatially organized distribution that reflects the model’s learned discrimination criteria.

These qualitative observations suggest that the quality of the upstream feature extractor is associated with the spatial interpretability of MIL attention mechanisms. Under weak features, attention maps—regardless of the sophistication of the aggregation architecture—lack the signal gradient necessary to produce spatially meaningful patterns. Under strong features, the same aggregation mechanism generates attention distributions that exhibit clear correspondence with pathologically relevant tissue structures. This finding has important implications for interpretability research: evaluations of attention-based spatial interpretability should explicitly account for feature extractor quality, as conclusions drawn from weak-feature attention maps may fundamentally mischaracterize the interpretive capacity of the aggregation architecture itself.

Quantitative spatial alignment analysis. To complement the qualitative observations with polarity-agnostic quantitative evidence, we computed the Normalized Attention Alignment Ratio (NAAR = mean attention within annotated region/mean attention over the entire valid tissue area) for each annotated WSI. Values close to 1.0 indicate spatial distributions indistinguishable from random, while |NAAR − 1.0| measures the deviation from random in a manner agnostic to whether the annotated region depicts malignant or non-malignant tissue—a property necessitated by the mixed annotation design in which some slides delineated malignant epithelium and others highlighted normal/stromal regions.

Table 7 summarizes the quantitative spatial alignment results. As shown in Panel B, mean |NAAR − 1.0| was 0.080 (Phikon-v2), 0.094 (UNI), and 0.113 (CTransPath). Paired Wilcoxon tests indicated that both CTransPath and UNI produced significantly larger deviations from random than Phikon-v2 (CTransPath vs. Phikon-v2: Δ = +0.032, *p* = 0.008; UNI vs. Phikon-v2: Δ = +0.017, *p* = 0.032), while CTransPath and UNI did not differ significantly (Δ = −0.015, *p* = 0.171). The secondary metric—Attention Mass Ratio at the top 25% threshold—showed the same pattern (|AMR − baseline| of 0.019, 0.031, and 0.033 for Phikon-v2, UNI, and CTransPath, respectively), with consistent ordering across all three threshold levels.

These quantitative results support the qualitative observation that stronger foundation models—both CTransPath and UNI—generate spatially structured attention distributions departing from random, whereas Phikon-v2 attention maps remain close to random. The comparable spatial alignment between CTransPath and UNI, despite UNI’s substantially larger pretraining corpus (~100,000+ vs. ~32,000 WSIs), reinforces that pretraining objective and architectural design contribute as substantially as scale to feature quality.

## 4. Discussion

This study comparatively evaluated the three fundamental components of MIL-based WSI classification—feature extractor, aggregation strategy, and classification framework—for ovarian cancer subtype classification. Through 36 experimental conditions on 510 WSIs from the UBC-OCEAN dataset, we provide quantitative evidence for the relative importance of each component and identify practical guidelines for pipeline design.

### 4.1. Feature Extractor as the Primary Performance Driver

A central observation of this study is the markedly larger contribution of feature extractor choice relative to aggregation strategy. Across the three foundation models compared, cascade balanced accuracy with max pooling spanned 0.538 (Phikon-v2) to 0.925 (UNI), with CTransPath achieving 0.870 at an intermediate level (Δ = +0.387 between weakest and strongest, +0.055 between CTransPath and UNI). The best-to-worst aggregator gap within each feature space was substantially smaller (0.130 for Phikon-v2, 0.041 for CTransPath, 0.053 for UNI), yielding a feature-versus-aggregator variance ratio of approximately 7-to-1 between the extreme feature conditions. Importantly, the inclusion of CTransPath provides a direct test of whether pretraining scale alone determines feature quality. CTransPath, despite a smaller pretraining corpus (~32,000 WSIs) than Phikon-v2 (~58,000 WSIs), substantially outperformed Phikon-v2 across all metrics (cascade balanced accuracy +0.332 with max pooling, +0.349 with DTP-TransMIL) and approached UNI (~100,000+ WSIs). This pattern—where a smaller pretraining corpus yielded substantially stronger features—indicates that pretraining objective and architectural design contribute as substantially as scale to feature quality for ovarian subtype classification. Practitioners may therefore achieve substantial performance gains through informed feature extractor selection, at lower implementation complexity than developing increasingly sophisticated aggregation architectures.

The progressive shift in optimal aggregators across the three feature conditions provides further insight into the feature–aggregator interaction. With Phikon-v2, DTP-TransMIL achieved the highest performance through its attention-guided patch selection and Transformer-based contextual modeling, suggesting that these mechanisms partially compensated for weak per-patch discriminability by integrating complementary signals across patches. With CTransPath features, DTP-TransMIL retained the top rank in Stage 1 (balanced accuracy 0.918) while max pooling led in Stage 2 (balanced accuracy 0.926), reflecting an intermediate regime in which attention-based mechanisms remained advantageous for the binary HGSC discrimination task but ceded ground to simpler aggregation in the four-class minority-subtype task. With UNI, where individual patches already encoded strong subtype-specific information, this compensatory mechanism became unnecessary, and the additional model parameters introduced overfitting on the 293-sample Stage 2 cohort. Max pooling, by contrast, benefited from its zero-parameter aggregation: the single most discriminative patch per feature dimension provided a sufficient and robust signal without the risk of learning spurious patterns. However, this interpretation should be qualified by the limited Stage 2 sample size (293 non-HGSC WSIs). With only 41 LGSC and 41 MC cases, attention-based models with learnable parameters are particularly susceptible to overfitting on minority classes. Max pooling’s advantage may therefore partly reflect a regularization benefit specific to small-cohort settings rather than an inherent architectural superiority; with larger training cohorts, attention-based methods may recover their competitive advantage. This interpretation is further supported by the substantially smaller aggregator performance gap under stronger features (0.041 for CTransPath and 0.053 for UNI, compared with 0.130 for Phikon-v2), indicating that stronger features substantially reduce the variance attributable to aggregation design.

This observation is consistent with the broader trend in computational pathology toward powerful foundation models. Breen et al. [22] evaluated 17 histopathology foundation models on 1864 WSIs and reported that model selection was the primary performance driver, with optimal cross-validation balanced accuracies of 73–83%. Our results extend this observation by explicitly quantifying the feature-versus-aggregator trade-off across three foundation models within a controlled experimental framework. While Breen et al. focused on foundation model comparison using a fixed aggregation method, our study demonstrates that even the simplest aggregator (max pooling) can match or exceed complex attention-based methods when paired with a sufficiently powerful feature extractor—a finding with immediate practical implications for resource-constrained research settings.

### 4.2. Clinical Value of Hierarchical Classification

The hierarchical two-stage cascade demonstrated a consistent and clinically meaningful improvement in HGSC recall compared to flat five-class classification. With UNI features and max pooling, the cascade achieved a 0.949 HGSC recall (206 of 217 cases detected) versus 0.876 for the flat baseline—identifying 16 additional HGSC patients who would otherwise have been misclassified. Given that HGSC identification directly determines eligibility for platinum-based chemotherapy and PARP inhibitor therapy [23], this improvement has direct therapeutic implications.

The majority suppression phenomenon—whereby aggressive class balancing paradoxically reduces the detection of the most prevalent subtype—represents a recognized but insufficiently documented challenge in imbalanced medical image classification [19]. In our flat five-class setting, simultaneously upweighting LGSC and MC (each representing 8.0% of the cohort) effectively penalized HGSC predictions, leading to the loss of 27 HGSC cases. The hierarchical design resolves this tension by isolating HGSC detection as an independent binary objective in Stage 1, allowing Stage 2 to optimize exclusively for minority subtype discrimination without interfering with majority class performance.

Importantly, this benefit was architecture-agnostic: the HGSC recall improvement was observed across all six evaluated configurations spanning three feature extractors (Phikon-v2, CTransPath, UNI) and two aggregators (max pooling, DTP-TransMIL), with magnitudes ranging from +0.073 (UNI + max pooling) to +0.530 (Phikon-v2 + DTP-TransMIL). This consistency indicates that the hierarchical framework provides structural value independent of the specific aggregation method or feature extractor, making it a generalizable design principle applicable to any MIL pipeline encountering similar class imbalance. However, the hierarchical design introduces cascade error propagation: Stage 1 misclassifications cannot be corrected downstream. Improving Stage 1 sensitivity is therefore a critical priority for future development.

### 4.3. Feature Quality and Spatial Interpretability

Our spatial interpretability analysis revealed an important and previously underreported relationship between feature extractor quality and attention map reliability, supported by both qualitative observations (Figure 4) and polarity-agnostic quantitative metrics (Table 7). With Phikon-v2 features, the DTP-TransMIL attention mechanism—despite its sophisticated gated attention scoring—produced spatially diffuse maps indistinguishable from random noise (mean |NAAR − 1.0| = 0.080). With CTransPath and UNI features and the identical aggregation architecture, the same mechanism generated attention distributions that systematically avoided pathologist-annotated normal/stromal regions and concentrated on malignant tissue, with significantly larger deviations from random than Phikon-v2 (CTransPath: 0.113, *p* = 0.008; UNI: 0.094, *p* = 0.032; paired Wilcoxon).

This finding carries two implications. First, evaluations of MIL attention interpretability must control for feature quality. Studies concluding that attention mechanisms fail to localize diagnostically relevant regions [24] may be observing a feature limitation rather than an aggregation limitation. Our results demonstrate that the same aggregation architecture can produce both uninformative and pathologically meaningful attention patterns, depending solely on the upstream feature quality. Second, the comparable spatial alignment between CTransPath (~32 K pretraining WSIs) and UNI (~100 K+ WSIs)—both significantly outperforming Phikon-v2 in spatial structure—reinforces that pretraining objective and architectural design contribute as substantially as scale to attention reliability. The systematic avoidance of annotated normal/stromal regions in the CC case (Figure 4) suggests that strong foundation models may enable clinically useful spatial interpretability without requiring explicit spatial supervision. While these observations were derived from a single-rater annotation set, future work should incorporate exhaustive pixel-level annotations and multi-rater concordance studies.

### 4.4. Limitations

Several limitations warrant acknowledgment, primarily stemming from our focus on isolating methodological contributions. First, evaluation was restricted to a single multi-institutional dataset (UBC-OCEAN) without an external validation cohort. While our primary objective was to demonstrate the relative importance of pipeline components within a controlled setting, future work should validate these findings using leave-one-center-out cross-validation or independent clinical cohorts to confirm cross-institutional generalizability, building upon recent advances in domain-generalized frameworks for ovarian cancer histology [25].

Second, the feature extractors were used in frozen configurations without fine-tuning. This design choice was deliberate to prevent overfitting on the relatively small 510-WSI cohort and to isolate the impact of pretrained feature quality independent of task-specific optimization. However, end-to-end fine-tuning may alter the relative performance patterns observed, particularly by potentially reducing the gap between feature extractors.

Third, while we extended the spatial interpretability analysis to a polarity-agnostic quantitative framework (NAAR and AMR; Section 3.4, Table 7), the underlying annotations were performed by a single observer with mixed semantic targets. Inter-observer agreement was therefore not assessed, and pixel-level pathologically exhaustive annotations remain a target for future research using multi-rater concordance studies.

Fourth, the five-fold cross-validation design, while standard for the dataset size, limits statistical power for pairwise comparisons. The minimum achievable raw *p*-value is 0.031, which after Holm–Bonferroni correction for five simultaneous tests becomes 0.156—exceeding the conventional significance threshold regardless of the observed effect magnitude. We addressed this limitation by reporting Cohen’s d as a complementary effect size measure, consistent with established recommendations for small-sample non-parametric testing [26].

Fifth, while we extended the comparison to three pathology foundation models (Phikon-v2, CTransPath, and UNI), the discriminative-power and pretraining-objective space of pathology foundation models remains substantially larger. Additional architectures such as Virchow [13] may yield different aggregator ranking patterns. Similarly, the DTP-TransMIL hyperparameters (e.g., the top 25% selection ratio, *K_min_* and *K_max_*) were selected based on preliminary experiments rather than exhaustive grid search; systematic optimization may further improve its relative standing.

## 5. Conclusions

This study provides three principal findings for MIL-based ovarian cancer subtype classification in whole slide images.

First, within this comparison of three pathology foundation models, feature extractor choice contributed substantially more variance to cascade balanced accuracy than aggregator selection. Cascade balanced accuracy with max pooling spanned 0.538 (Phikon-v2) to 0.925 (UNI), with CTransPath achieving 0.870, while the best-to-worst aggregator gap within each feature space was substantially smaller (0.041–0.130). Notably, CTransPath—pretrained on a smaller corpus (~32,000 WSIs) than Phikon-v2 (~58,000 WSIs)—substantially outperformed Phikon-v2, indicating that pretraining objective and architecture contribute as substantially as scale to feature quality. With UNI features, even the simplest aggregator (max pooling) surpassed all attention-based methods, achieving a Stage 2 balanced accuracy of 0.957. Conversely, with Phikon-v2 features, architecturally complex methods (DTP-TransMIL) provided the greatest compensatory benefit. This inverse relationship between feature quality and aggregator complexity suggests that practitioners may benefit from prioritizing feature extractor evaluation before investing in sophisticated aggregation architectures.

Second, the hierarchical two-stage cascade consistently improved HGSC recall over flat classification across all six evaluated configurations (three feature extractors × two aggregators), with improvements ranging from +0.073 (UNI + max pooling) to +0.530 (Phikon-v2 + DTP-TransMIL). The optimal configuration achieved 0.949 HGSC recall with UNI + max pooling (206 of 217 cases detected, 11 missed). This consistency confirms that the benefit arises from the structural decomposition itself rather than any specific model characteristic. Given that HGSC identification informs eligibility for platinum-based chemotherapy and PARP inhibitors, the clinical value of this improvement is substantial.

Third, feature quality was associated with the spatial interpretability of attention mechanisms across all three foundation models compared. Both qualitative attention map analysis and polarity-agnostic quantitative metrics (NAAR, AMR; Table 7) revealed that Phikon-v2-based attention maps were close to random, while CTransPath and UNI generated significantly more spatially structured distributions (paired Wilcoxon *p* = 0.008 and *p* = 0.032, respectively). This finding implies that conclusions about MIL spatial interpretability should always be qualified by the underlying feature quality.

The optimal pipeline configuration identified—UNI features with max pooling in a hierarchical cascade—achieved a cascade balanced accuracy of 0.925, with per-class recalls exceeding 0.86 for all five subtypes. The architectural patterns observed are consistent within UBC-OCEAN: the hierarchical decomposition is aggregator-agnostic and can be applied to any MIL pipeline, while the demonstrated feature–aggregator trade-off provides a practical decision framework for computational pathology pipeline design. Future work should validate these findings on external cohorts, extend the comparison to additional pathology foundation models such as Virchow [13] and other recent architectures, and investigate whether end-to-end fine-tuning alters the feature–aggregator interaction patterns observed under frozen feature conditions.

## Figures and Tables

**Table 1:** Dataset characteristics of the 510 WSIs used in this study.

Subtype	N (%)	Patches (min)	Patches (max)	Patches (Median)	Patches (Mean ± SD)
HGSC	217 (42.5%)	344	29,057	6838	8531 ± 6209
EC	118 (23.1%)	459	34,161	8792	10,163 ± 6825
CC	93 (18.2%)	519	28,525	8643	10,415 ± 6724
LGSC	41 (8.0%)	228	24,449	4974	7406 ± 6104
MC	41 (8.0%)	1495	34,412	12,104	13,739 ± 7070
Total	510 (100%)	228	34,412	7864	9593 ± 6668

Patches were extracted at 256 × 256 pixels with non-overlapping tiling. Only patches with a tissue area exceeding 20% were retained. Abbreviations: HGSC, high-grade serous carcinoma; EC, endometrioid carcinoma; CC, clear cell carcinoma; LGSC, low-grade serous carcinoma; MC, mucinous carcinoma; SD, standard deviation.

**Table 2:** Computational complexity of each aggregator. Parameters are reported for the Stage 2 configuration (n_classes = 4, input_dim = 1024 from UNI). FLOPs and inference time were measured at N = 7864 patches per slide (the cohort median; see Table 1). Inference time is the median of 50 forward passes on a single NVIDIA RTX A4000 (16 GB) with FP16 mixed precision; standard deviations were under 5% of the median in all cases.

Aggregator	Parameters	GFLOPs	Inference (ms)
Mean pooling	657,156	8.254	0.40
Max pooling	657,156	8.254	0.44
ABMIL	789,765	12.389	0.73
CLAM-SB	793,869	12.389	0.73
DSMIL	792,588	12.434	1.28
DTP-TransMIL	4,996,869	36.540	2.26

**Table 3:** Aggregator performance comparison across three feature extractors (five-fold cross-validation, mean ± SD). Stage 1: HGSC vs. non-HGSC binary classification. Stage 2: EC/CC/LGSC/MC four-class classification. Bold values indicate the best performance per feature extractor and stage.

Feature Extractor	Aggregator	S1 AUROC	S1 BalAcc	S2 AUROC	S2 BalAcc
Phikon-v2	Mean pooling	0.660 ± 0.040	0.648 ± 0.028	0.756 ± 0.042	0.504 ± 0.071
	Max pooling	0.718 ± 0.036	0.706 ± 0.019	0.759 ± 0.014	0.593 ± 0.041
	ABMIL	0.666 ± 0.046	0.670 ± 0.039	0.772 ± 0.017	0.613 ± 0.028
	CLAM-SB	0.675 ± 0.077	0.641 ± 0.080	0.777 ± 0.019	0.588 ± 0.027
	DSMIL	0.642 ± 0.043	0.644 ± 0.047	0.768 ± 0.038	0.573 ± 0.046
	DTP-TransMIL	0.715 ± 0.071	0.696 ± 0.054	0.793 ± 0.043	0.634 ± 0.056
CTransPath	Mean pooling	0.939 ± 0.023	0.859 ± 0.023	0.954 ± 0.019	0.885 ± 0.048
	Max pooling	0.963 ± 0.015	0.890 ± 0.027	0.968 ± 0.020	0.926 ± 0.050
	ABMIL	0.954 ± 0.021	0.861 ± 0.033	0.966 ± 0.023	0.901 ± 0.052
	CLAM-SB	0.950 ± 0.017	0.872 ± 0.015	0.966 ± 0.031	0.901 ± 0.059
	DSMIL	0.948 ± 0.018	0.861 ± 0.046	0.970 ± 0.030	0.905 ± 0.059
	DTP-TransMIL	0.966 ± 0.015	0.918 ± 0.016	0.965 ± 0.025	0.901 ± 0.046
UNI	Mean pooling	0.971 ± 0.014	0.904 ± 0.030	0.975 ± 0.018	0.904 ± 0.041
	Max pooling	0.982 ± 0.013	0.913 ± 0.032	0.985 ± 0.017	0.957 ± 0.044
	ABMIL	0.979 ± 0.013	0.907 ± 0.020	0.983 ± 0.016	0.930 ± 0.047
	CLAM-SB	0.978 ± 0.014	0.908 ± 0.022	0.987 ± 0.012	0.933 ± 0.051
	DSMIL	0.979 ± 0.013	0.914 ± 0.005	0.986 ± 0.016	0.945 ± 0.039
	DTP-TransMIL	0.979 ± 0.010	0.931 ± 0.027	0.961 ± 0.020	0.914 ± 0.052

Abbreviations: S1, Stage 1; S2, Stage 2; AUROC, area under the receiver operating characteristic curve; BalAcc, balanced accuracy. All experiments used identical data splits, loss function, and training protocol.

**Table 4:** Pairwise statistical comparison of max pooling versus each baseline aggregator (Stage 2, balanced accuracy, UNI features). One-sided Wilcoxon signed-rank test (*n* = 5 folds; minimum achievable *p* = 0.031). *p*-values adjusted using the Holm–Bonferroni method for five simultaneous comparisons.

Comparison	Δ BalAcc	Raw *p*	Holm-adj *p*	Cohen’s d	Effect Size
Max pooling vs. Mean pooling	+0.053	0.031	0.156	4.91	Very large
Max pooling vs. DTP-TransMIL	+0.043	0.031	0.062	2.88	Very large
Max pooling vs. CLAM-SB	+0.024	0.031	0.094	1.60	Large
Max pooling vs. ABMIL	+0.027	0.031	0.125	1.33	Large
Max pooling vs. DSMIL	+0.012	0.219	0.219	0.55	Medium

Δ: mean difference (max pooling − baseline). Cohen’s d: standardized paired effect size. Conventional thresholds: small (d = 0.2), medium (d = 0.5), large (d = 0.8).

**Table 5:** Hierarchical cascade versus flat five-class classification across feature extractors and aggregators. Cascade results use out-of-fold predictions with Youden’s J threshold optimization per fold. Bold values indicate the best performance per metric.

Feature Extractor	Aggregator	Strategy	BalAcc	HGSC Recall	HGSC Missed, N (%)
Phikon-v2	Max pooling	Cascade	0.538	0.719	61 (28.1%)
		Flat	0.494	0.470	115 (53.0%)
	DTP-TransMIL	Cascade	0.507	0.654	75 (34.6%)
		Flat	0.468	0.124	190 (87.6%)
CTransPath	Max pooling	Cascade	0.870	0.917	18 (8.3%)
		Flat	0.876	0.774	49 (22.6%)
	DTP-TransMIL	Cascade	0.856	0.903	21 (9.7%)
		Flat	0.842	0.788	46 (21.2%)
UNI	Max pooling	Cascade	0.925	0.949	11 (5.1%)
		Flat	0.915	0.876	27 (12.4%)
	DTP-TransMIL	Cascade	0.865	0.945	12 (5.5%)
		Flat	0.882	0.871	28 (12.9%)

BalAcc: balanced accuracy computed across all five subtypes. HGSC Recall: proportion of HGSC cases correctly identified. HGSC Missed: number and percentage of HGSC cases misclassified as non-HGSC by Stage 1 (cascade) or as other subtypes (flat).

**Table 6:** Per-class classification performance of the optimal pipeline configuration (UNI + max pooling, hierarchical cascade). Values represent out-of-fold predictions aggregated across all five folds.

Subtype	N	Precision	Recall	F1-Score
EC	118	0.895	0.864	0.879
CC	93	0.989	0.957	0.973
LGSC	41	0.860	0.902	0.881
MC	41	0.907	0.951	0.929
HGSC	217	0.936	0.949	0.943
Macro avg	510	0.917	0.925	0.921
Weighted avg	510	0.928	0.928	0.927

Stage 1 (HGSC vs. non-HGSC): 11 false negatives (HGSC missed), 14 false positives (non-HGSC misrouted as HGSC). Abbreviations: EC, endometrioid carcinoma; CC, clear cell carcinoma; LGSC, low-grade serous carcinoma; MC, mucinous carcinoma; HGSC, high-grade serous carcinoma.

**Table 7:** Quantitative spatial alignment between attention maps and pathologist annotations across three feature extractors. The Normalized Attention Alignment Ratio (NAAR) is the ratio of mean attention scores within annotated regions to mean attention scores over the entire valid tissue area; |NAAR − 1.0| is a polarity-agnostic measure of deviation from random spatial distribution. Attention Mass Ratio (AMR) is computed at the top 10%, 25%, and 50% attention thresholds; |AMR − baseline| measures deviation from the random expectation (baseline = annotated area fraction). Panel A reports per-feature summary statistics across all evaluable WSIs; Panel B reports paired Wilcoxon signed-rank tests on |NAAR − 1.0| restricted to WSIs evaluable for all three feature extractors. * *p* < 0.05; ** *p* < 0.01.

**(a) Per-Feature Summary**				
**Feature**	**NAAR**	**|NAAR − 1.0|**	**Median** **|NAAR − 1.0|**	**|AMR − Baseline| at Top 10%/25%/50%**
Phikon-v2	1.014 ± 0.165	0.080 ± 0.144	0.043	0.031/0.019/0.010
CTransPath	0.986 ± 0.166	0.113 ± 0.122	0.073	0.046/0.033/0.022
UNI	0.985 ± 0.146	0.094 ± 0.112	0.054	0.043/0.031/0.019
**(b) Paired Wilcoxon tests (|NAAR − 1.0|)**				
**Comparison**	**Δ**	**Wilcoxon W**	***p*-value**	**Significance**
CTransPath vs. Phikon-v2	+0.032	3667	0.008	**
UNI vs. Phikon-v2	+0.017	3906	0.032	*
CTransPath vs. UNI	−0.015	4277	0.171	n.s.

## Data Availability

The UBC-OCEAN dataset used in this study is publicly available at https://www.kaggle.com/competitions/UBC-OCEAN (accessed on 21 March 2026). The trained model weights and analysis code are not publicly deposited but are available from the corresponding author upon reasonable request.
